# Immune-related response assessment during PD-1 inhibitor therapy in advanced non-small-cell lung cancer patients

**DOI:** 10.1186/s40425-016-0193-2

**Published:** 2016-12-20

**Authors:** Mizuki Nishino, Nikhil H. Ramaiya, Emily S. Chambers, Anika E. Adeni, Hiroto Hatabu, Pasi A. Jänne, F. Stephen Hodi, Mark M. Awad

**Affiliations:** 1 Department of Radiology, Brigham and Women’s Hospital and Dana-Farber Cancer Institute, 450 Brookline Ave., Boston, MA 02215 USA; 2 Department of Medical Oncology and Department of Medicine Dana-Farber Cancer Institute and Brigham and Women’s Hospital, 450 Brookline Ave., Boston, MA 02215 USA

**Keywords:** PD-1 inhibitor, Immunotherapy, Non-small-cell lung cancer, Computed tomography, Tumor response evaluation

## Abstract

**Background:**

Tumor response characteristics using immune-related RECIST1.1 (irRECIST1.1) in advanced non-small-cell lung cancer (NSCLC) patients treated with nivolumab monotherapy in the clinical setting have not been previously described with a direct comparison with the assessments according to the conventional RECIST1.1.

**Methods:**

Fifty-six advanced NSCLC patients treated with nivolumab monotherapy after its Food and Drug Administration (FDA) approval were retrospectively studied. Tumor burden was quantified on serial CT scans during therapy using irRECIST1.1, which uses unidimensional measurements and includes new lesion measurements in total tumor burden. Response assessments by irRECIST1.1 were compared with assessments by RECIST1.1. Responses of individual lesions in different organs were also compared.

**Results:**

Tumor burden change at best overall response ranged from −66.8 to +278.1% (median: +3.9%). Response rate was 14% (8/56; 8 partial responses, 0 complete responses) by irRECIST1.1 and by RECIST1.1. Time-to-progression (TTP) by irRECIST1.1 was longer than TTP by RECIST1.1 (median TTP: not reached vs. 1.9 months, respectively). No patients experienced pseudoprogression during the study. Among 128 target lesions, the lesion-based size change at best response differed significantly across different organs, with adrenal lesions and lymph nodes having greater size decrease, followed by lung, while liver and other miscellaneous lesions had lesser degree of size decrease (*p* = 0.002).

**Conclusions:**

Immune-related response evaluations using irRECIST1.1 in advanced NSCLC patients treated with nivolumab resulted in the identical response rate and longer TTP compared to RECIST1.1. No pseudoprogression cases were observed during the study. Adrenal lesions and lymph nodes were more responsive and liver lesions were less responsive to nivolumab.

**Electronic supplementary material:**

The online version of this article (doi:10.1186/s40425-016-0193-2) contains supplementary material, which is available to authorized users.

## Background

Programmed cell death (PD)-1 immune checkpoint inhibitors have emerged as promising treatment options for multiple cancer types [1–11]. Two PD-1 inhibitors, nivolumab and pembrolizumab, were recently approved for treatment of advanced non-small-cell lung cancer (NSCLC), resulting in widespread use in these agents in the clinical setting. These agents have shown response rates ranging from 14.5–19.4% in recent NSCLC trials [4, 8, 9, 12, 13]. In two phase 3 studies of nivolumab for NSCLC, a higher response rate and longer overall survival (OS) were observed in nivolumab-treated patients compared to docetaxel-treated patients, both in squamous NSCLC [14] and non-squamous NSCLC [12]. Another recent phase 3 study of pembrolizumab in advanced NSCLC also showed a higher response rate and longer OS in patients treated with pembrolizumab compared to those treated with docetaxel [13].

Traditionally, the anti-tumor activity of a therapeutic agent is assessed using the Response Evaluation Criteria in Solid Tumors (RECIST) guidelines, originally published in 2000 and revised in 2009 as RECIST1.1, which utilizes unidimensional, longest diameter measurements to quantify tumor burden [15–17]. Response assessment by RECIST1.1 is frequently used as a primary endpoint in many clinical trials. According to RECIST1.1, patients are categorized as having progressive disease (PD) when their tumor burden increases above 20% and 5 mm comparing to baseline, or when the appearance of new lesions is noted [16, 17]. However, in patients treated with immune-checkpoint inhibitors, unique radiographic response patterns have been noted, such as a response after an initial increase of tumor burden or a reduction in tumor burden during or after the appearance of new lesions, which would otherwise be classified as PD by RECIST1.1 [18–20]. To capture these unconventional response patterns termed “pseudoprogression”, a novel set of criteria, immune-related response criteria (irRC) was proposed in 2009 [20]. The key features of irRC are 1) inclusion of new lesion measurements to the total tumor burden and 2) requirement of confirmation of PD on two consecutive scans at least 4 weeks apart [18–20]. Subsequently, irRC has been applied in trials of immune checkpoint inhibitors in NSCLC and melanoma to define trial endpoints [21–23].

Although irRC has introduced the novel important concepts of immune-related response assessment, there are issues that remain to be solved. A major methodological issue is that irRC uses bidimensional measurements derived from a product of the longest diameter and the longest perpendicular diameter as proposed in WHO criteria back in 1981 [24], while most trials in solid tumors over the past decade have been based on RECIST and thus used unidimensional measurements [18, 20]. It is therefore difficult to directly compare the results obtained by using irRC versus RECIST, within the same trial cohorts and across the different trials; some differences may be simply due to the difference from unidimensional versus bidimensional measures, and may not reflect the difference in tumor burden dynamics such as the occurrence of immune-related tumor response phenomena or the different magnitude of responses to different agents [19, 25, 26].

To overcome this methodological issue of immune-related response evaluations, a prior study of ipilimumab in melanoma assessed unidimensional irRC utilizing the longest diameter measurements as in RECIST, and demonstrated that unidimensional irRC provides highly concordant response assessment compared to bidimensional irRC with less measurement variability [26]. Another study further incorporated the revised features of RECIST1.1 into unidimensional irRC, such as the decrease in the number of target lesions and the use of short axis measurements for lymph nodes, and demonstrated high concordance of response assessments [25], proposing a direction toward immune-related RECIST1.1 (irRECIST1.1) to evaluate anti-tumor activity of immune-checkpoint inhibitor therapy in solid tumors [19, 25].

Although there is an increasing recognition of the modified strategies such as irRC and irRECIST1.1 that are specifically designed to address the immune-related response phenomena, most current immunotherapy trials rely on conventional RECIST1.1 to obtain standardized endpoints that have been used as the basis for FDA approval. Of note, immune-related response evaluations in comparison with RECIST1.1 are not described in the recent trials of PD-1 inhibitors in NSCLC, and there is very limited data about the frequency of immune-related response phenomena such as pseudoprogression in advanced NSCLC patients [27]. Lack of such information may lead to challenges in the treatment decisions in patients with tumor burden increase during PD-1 inhibitor therapy in the clinical setting, where oncologists need to decide if they continue therapy for the possibility of pseudoprogression, or change to alternate therapies for the possibility of true progression. The challenges can be particularly significant when FDA-approved commercial agents are administered, as treatment discontinuation decisions are not guided by specific trial protocols that can help determine which criteria to use in tumor burden assessments and when to consider terminating therapy. This unmet clinical need has provided a motivation to systematically investigate tumor response characteristics of advanced NSCLC patients treated with commercially prescribed PD-1 inhibitor therapy, as an initial step to further address this emerging challenge in lung cancer treatment.

The purpose of the present study is to evaluate tumor response characteristics using irRECIST1.1 in advanced NSCLC patients treated with nivolumab in the clinical setting after its FDA approval, and compare the results with those obtained by the conventional RECIST1.1.

## Results and discussion

The demographics and disease characteristics of the 56 patients are summarized in Table 1. Median time on therapy was 3.8 months (95%CI: 2.6–4.2). Median follow-up time was 3.8 months (95%CI: 3.2–4.2). Twenty-one patients (38%) were still on nivolumab therapy at the time of analysis.

**Table 1:** Demographics and clinical characteristics of the 56 patients, categorized by responders and non-responders

Characteristics		All patients (*n* = 56)	Responders^a^ (*n* = 8)	Non-responders (*n* = 48)	*P* value
Sex	Male	31 (55%)	5	26	0.72
	Female	25 (45%)	3	22	
Age (years)	Median (range)	65 (43–91)	62 (44–74)	65 (43–91)	0.29
Race	White	49 (88%)	8	41	1.00
	Black	3 (5%)	0	3	
	Asian	2 (3.5%)	0	2	
	Other	2 (3.5%)	0	2	
Smoking	Never	10 (18%)	1	9	1.00
	Former	27 (48%)	4	23	
	Current	19 (34%)	3	16	
Histology	Adenocarcinoma	40 (71%)	6	34	0.59
	Squamous cell carcinoma	9 (16%)	2	7	
	NSCLC NOS^b^	7 (13%)	0	7	

^a^irRECIST1.1 and RECIST1.1 have resulted in the same 8 responders

^b^NSCLC NOS = non-small-cell carcinoma not otherwise specified

### Response assessment by irRECIST and RECIST1.1

**Fig. 1:**
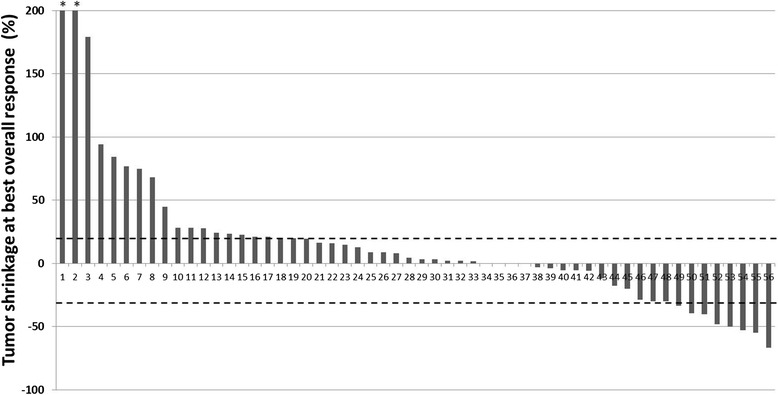
Waterfall plot of tumor burden change at best overall response in 56 patients. Two patients marked by * had greater than 200% (+219% and +278%). Dashed lines represent the thresholds for partial response (−30%) and progressive disease (+20%)

**Fig. 2:**
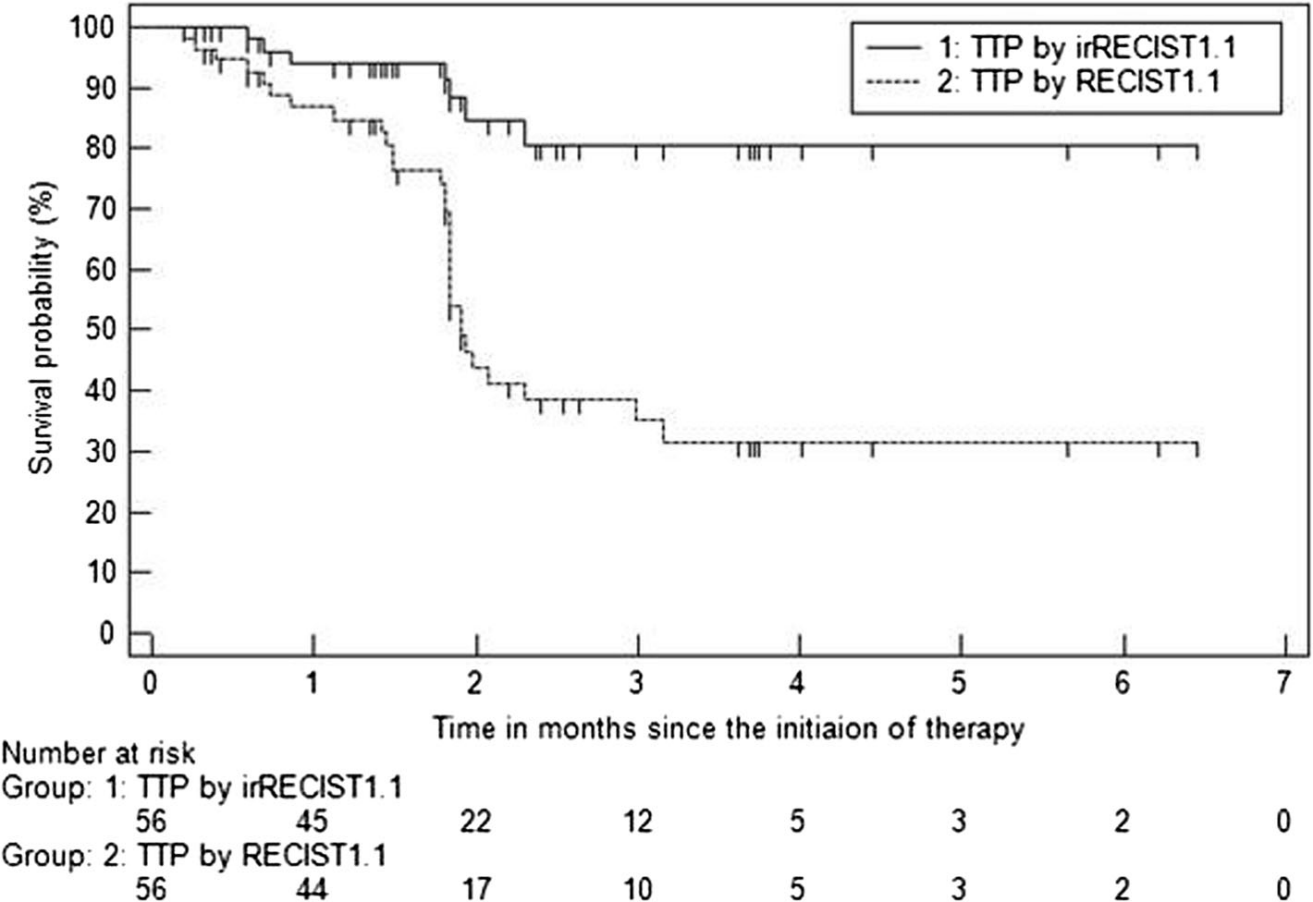
Time to progression by irRECIST and RECIST1.1

Tumor burden changes in reference to baseline (%) at the time point of best overall response in 56 patients ranged from −66.8 to +278.1% (median: +3.9%) (Fig. 1). Response rate was 14% (8/56; 8 PR, 0 CR), which was identical between irRECIST1.1 and RECIST1.1. Discordance of BOR between irRECIST1.1 and RECIST1.1 was noted in 20 patients, in which BOR was SD by irRECIST1.1 but was PD by RECIST1.1. Among these 20 patients, 10 did not have a confirmation of PD. Of these, 8 patients had no subsequent scans, and 2 patients had one subsequent scan again demonstrating progression; however these scans were performed less than 4 weeks from the previous scan, and no further scans were performed thereafter. In the remaining 10 patients, new lesions were included in the measurements by irRECIST1.1 rather than immediately defining PD. TTP by irRECIST1.1 (irTTP) was longer than TTP by RECIST1.1 (median TTP: not reached vs 1.9 months, respectively) (Fig. 2). There were no significant differences in demographics or disease characteristics between responders and non-responders (*p* > 0.29) (Table 1).

**Fig. 3:**
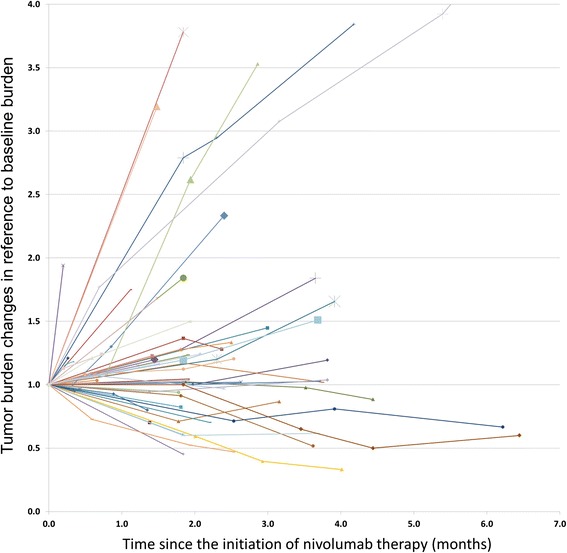
Spider plot of tumor burden changes during nivolumab therapy using irRECIST. Longitudinal changes of tumor burden during therapy are shown in reference to baseline, showing baseline tumor burden as 1.0. The scan time points with the appearance of new target lesions are indicated by larger markers

The tumor burden changes during nivolumab therapy using irRECIST1.1 are demonstrated in the spider plot (Fig. 3). None of the patients in this study demonstrated pseudoprogression at the time of analysis, defined as initial increase of tumor burden followed by subsequent response.

### New lesions during nivolumab therapy

Eighteen patients (18/56; 32%) developed new lesions during therapy. Among them, 9 patients developed new target and non-target new lesions, 2 patients developed new target lesions alone, and 7 patients developed new non-target lesions alone. In the 11 patients with new target lesions, the number of new target lesions ranged from 1–4 per patient (median: 2). The most common location of new target lesions was the liver, noted in 7 patients, followed by lymph nodes (3 patients), adrenal gland (2 patients), and lung (1 patient). Among new non-target lesions, liver was the most common site (noted in 7 patients), followed by lung (3 patients), lymph nodes (3 patients), brain (2 patients), bone (2 patients), and bowel (1 patient).

At the time of appearance of new lesions, no patients met the criteria for partial response by irRECIST. However, 8 of the 18 patients with new lesions had SD (with less than 20% increase) based on the irRECIST measurements at the time of appearance of new lesions; of these, 6 patients had new non-target lesions only, and 2 patients had both new target and non-target lesions. In the remaining 10 patients, the irRECIST measurements showed PD (with at least 20% increase); of these, one patient had new non-target lesions alone, 2 patients had new target lesions alone, and 7 patients had new target and non-target lesions.

Of the 18 patients, 5 patients had follow-up scans after the appearance of new target lesions. None of the new lesions showed response during the follow-up period of this study. In 4 patients, new lesions progressed with ≥20% and 5 mm increase on their first follow-up scan comparing to the size when they appeared; in one patient, new lesions showed stable disease on the first follow-up scan, followed by disease progression on the subsequent scan. Additional 3 patients with new non-target lesion alone had follow-up scans, which showed “stable disease” (non-CR, non-PD) for these lesions. Among the remaining 10 patients who had no follow-up scans after the appearance of new lesions, nivolumab therapy was ended in 8 patients while 2 patients were still on therapy at the time of analyses.

### Lesion-based response assessment

A total of 128 target lesions were present in the cohort of 56 patients at baseline (median 2 lesions per patient), including 56 lung lesions, 31 lymph nodes, 16 liver lesions, 14 adrenal lesions, and 11 other lesions in miscellaneous locations. Thirty-five patients (62.5%) had more than one lesion in the same organ, and 4 of them had multiple lesions in two organs. The most common organ with multiple target lesions was lung, noted in 16 patients, followed by lymph (10 patients), liver (7 patients), adrenal gland (4 patients), and other (2 patients) (Additional file 1).

**Fig. 4:**
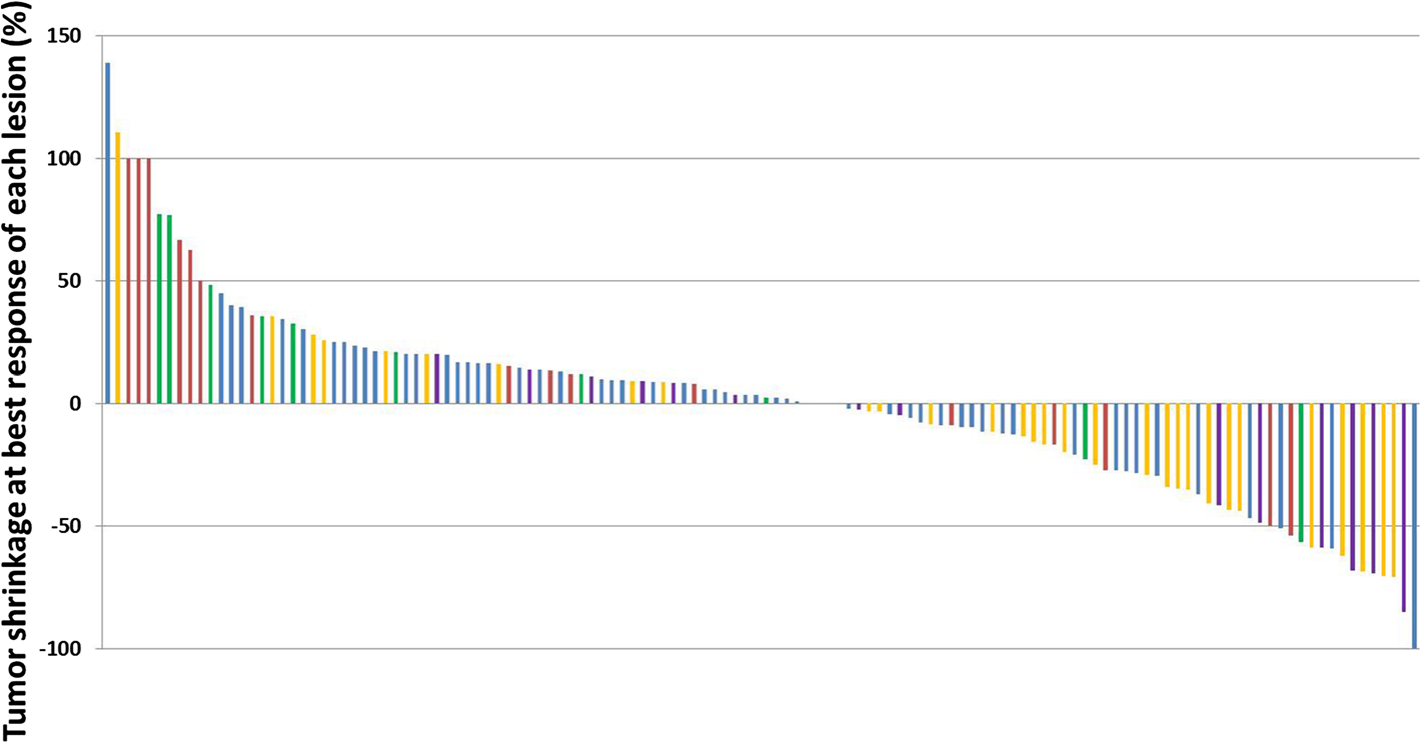
Waterfall plot of lesion-based tumor size change at best response of 128 target lesions classified by the organs. The graph shows changes of lung lesions in blue bars, lymph nodes in yellow, adrenal lesions in purple, liver lesions in red, and other miscellaneous lesions in green bars

**Fig. 5:**
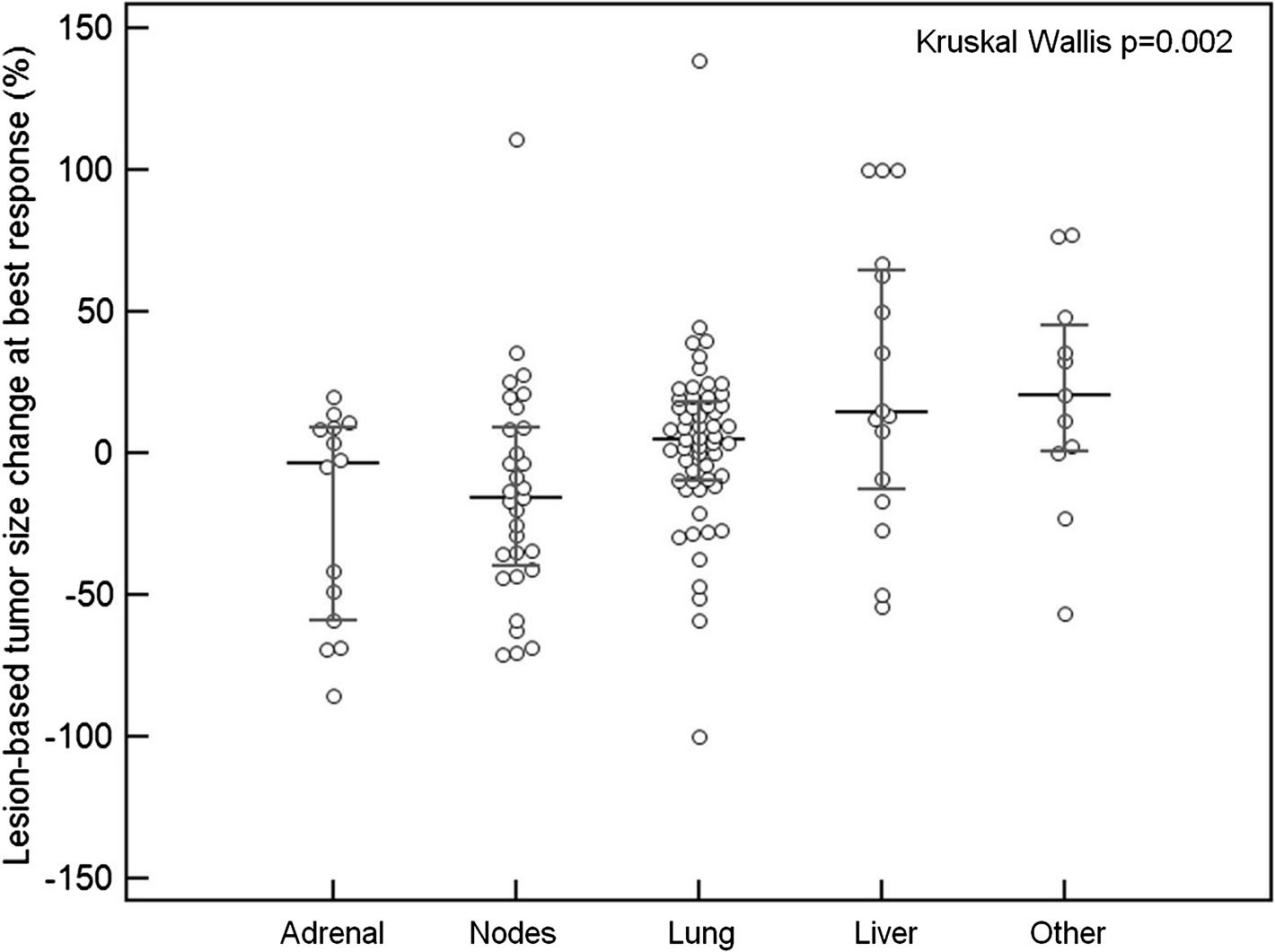
Lesion-based tumor size change at best response of 128 target lesions classified by the organs. The black horizontal line shows a median value for the lesion-based shrinkage in each organ. The gray vertical line with horizontal bars at the upper and lower ends represents the 1^st^ and 3^rd^ quartiles

Lesion-based tumor size change (%) at best response of each lesion was significantly different across the organ categories(Kruskal-Wallis *p* = 0.002). Adrenal lesions and lymph nodes had greater shrinkage, followed by lung, while liver and miscellaneous lesions had less shrinkage (Figs. 4, and 5). The response rates also significantly differed among the lesion groups according to the location (Fisher *p* = 0.004). The response rate was highest in adrenal gland lesions (6/14; 43%) followed by lymph nodes (11/31; 35%), while the rates were lower in lung (5/56; 9%), liver (2/16; 12%), and miscellaneous (1/11; 9%) lesions.

## Discussion

The present study describes the initial results of tumor response characteristics using irRECIST1.1 among advanced NSCLC patients treated with commercially-prescribed nivolumab in the clinical setting, and provides a direct comparison of the assessment results between irRECIST1.1 and RECIST1.1. The response rate in this cohort was 14% both by irRECIST1.1 and by RECIST1.1. No patient experienced pseudoprogression during the study period. Lesion-based assessment showed significant differences of responses across organs, with adrenal lesions and lymph nodes being more responsive and liver lesions being less responsive to therapy. To our knowledge, this is the first study that demonstrated the organ-specific tumor responses in patients treated with immune checkpoint inhibitors.

The overall response rate of 14% in the present study is similar to prior clinical trials of nivolumab or pembrolizumab in NSCLC, where response rates of 14.5–19.4% were reported [4, 8, 9, 12]. All responders in the present cohort had PR and no patient achieved CR; this is also similar to clinical trial results, where CR was noted in 0 of 117 nivolumab-treated squamous NSCLC patients [4], in 0.8% among 495 pembrolizumab-treated NSCLC patients [9], and 1% among 292 nivolumab-treated non- squamous NSCLC patients [12]. Tumor burden change at BOR per patient had a wide range (−66.8 to +278.1%) with a median of 3.9% in the present study, indicating a wide range of responses and an apparent heterogeneity of sensitivity to PD-1 inhibitors among advanced NCCLC population as noted in the trials [4, 8, 12]. Demographics and clinical characteristics including histology and smoking history showed no association with response to nivolumab. In the recent two trials of PD-1 inhibitor, the response rate was similar between squamous and nonsquamous NSCLC [8, 9], which is consistent with our results. Smoking history was associated with a higher rate of response to nivolumab in a phase 1 study [8]; however, such observation was not noted in our cohort, likely due to our small sample size. Other molecular markers such as PD-L1 positivity of tumor tissue were not available in most patients in this clinical retrospective cohort.

Head-to-head comparisons between immune-related response evaluations and the conventional RECIST assessments are lacking in most of the trials of immune-checkpoint inhibitor therapy, as recently described by Chiou et al. [27]. Therefore, providing such comparisons was one of the major goals of the present study. Assessment of PR was fully concordant between irRECIST1.1 and RECIST1.1, in this cohort with no cases of pseudoprogression. The discordance of BOR assessment between two criteria was only noted between SD and PD, either because of the requirement of confirmation for PD or the inclusion of new lesions in the total tumor burden by irRECIST1.1, which contributed equally to the discordance of BOR. These two features of irRECIST1.1 also contributed to longer TTP by irRECIST1.1 (irTTP) compared to TTP by RECIST1.1, which is an expected consequence of immune-related response evaluations as reported in a prior study of melanoma patients treated with ipilimumab [25]. Median TTP by RECIST1.1 was 1.9 months in the present cohort; in spite of the relatively short follow-up period of the present study, the result is overall similar to the previous studies in the trial cohorts reporting median PFS of 1.9-3.7 months based on RECIST [4, 8, 9, 12]. The present study focused on TTP, rather than PFS, as its major purpose was the differences derived from two tumor response criteria, which TTP reflects most accurately and is not affected by death as an event.

Pseudoprogression, or initial progression with tumor burden increase followed by subsequent response, is a challenging phenomenon during immune checkpoint inhibitor therapy. None of the patients in the present study experienced pseudoprogression during the treatment period of the study, which can be due to the small number of patients studied in a relatively short period of time during this initial clinical experience. In a phase 1 study of nivolumab in advanced NSCLC, 5% (6/129) of the patients experienced pseudoprogression, which were within 20 weeks since initiation of therapy [8]. On the other hand, a recent study et al. in 44 NSCLC patients treated with trials of PD-1 and PD-L1 inhibitors, 4 patients (9%), all of whom received PD-L1 inhibitors, had experienced pseudoprogression at 3 months of therapy [28]. Further investigations are needed in larger cohorts to determine the exact incidence of pseudoprogression in advanced NSCLC patients subclassified according to the types of immune-checkpoint inhibitors used in the treatment regimen.

The appearance of new lesions presents another management dilemma during immune-checkpoint inhibitor therapy because it may indicate true progression or pseudoprogression. In the present study, one-third of patients developed new lesions, most commonly in the liver. Though no patients showed partial response based on irRECIST measurements at the time of appearance of new lesions, 44% of the patients (8/18) had measureable tumor burden changes within the range of SD. Notably, 2 of these patients had new target lesions and had SD even though the measurements of these new lesions were added to the total tumor burden. These observations are indicative of the merit of the use of irRECIST for detection of stable disease in the setting of appearance of new lesions.

Most patients with follow-up scan after the appearance of new lesions showed progression of new lesions, and none of the new lesions subsequently responded during therapy. Though very few reports describe the detailed locations and behaviors of new lesions during immune-checkpoint inhibitor therapy, in a recent study by Caramella et al., 3 of the 4 patients with pseudoprogression had new lesions in the chest (2 in the lung and one in the mediastinum) during PD-L1 inhibitor therapy [28]. Precise descriptions of new lesion location and its behavior during therapy are needed in future studies to further understand the phenomenon of pseudoprogression in the setting of new lesions, and to differentiate it from true progression for better treatment guidance.

Lesion-based response assessment resulted in notable differences in the response rate and the degree of tumor size decrease across anatomic organs of the lesions. Adrenal lesions and lymph nodes are more responsive with a greater tumor size decrease, while liver lesions were less responsive with less size decrease. Although the exact mechanisms of this observation are uncertain, it may indicate the impact of different tumor microenvironments in different organs on immune-related tumor response. The liver has an intrinsic immune suppressive microenvironment [29, 30], which may help tumors to escape from anti-tumor immune attacks during therapy, resulting in less tumor shrinkage. Lymph nodes as source organs of immune cells may be at least partly contribute to the observed greater response of lymph nodes as target lesions. Adrenal gland is one of the most common site of extrathoracic metastasis from NSCLC, and is also a major effector organ of hypothalamic–pituitary–adrenal (HPA) axis and is responsible for synthesis and action of cytokines [31, 32]. Therefore, the organ is known to have immune-modulating properties via activation of the HPA axis as well as via cell-cell mediated immune-adrenal interactions [31, 32]. The unique properties of the organ may be related to a greater degree of response in adrenal lesions.

To our knowledge, lesion-based immune-related responses across different organs have not been previously described in detail in NSCLC patients. Given the significant difference of responses across different organs noted in this study, lesion-based analysis may provide clinically significant information because the anatomic distribution of metastasis and selection of target lesions prior to therapy may affect the response outcome. Further investigations are needed to study the consistency of the observations in larger cohorts of NSCLC and to understand the underlying mechanisms. Notably, the results of a recent investigation by Ribas et al. in advanced or metastatic melanoma treated with pembrolizumab in phase 1b trials indicate that the response rate may be higher in patients with lung metastases (M1b disease) than in those with other visceral metastases (M1c disease) [33]. Another study in 337 melanoma patients treated with pembrolizumab or nivolumab demonstrated that patients with liver metastases were less likely to respond to treatment, while patients with lung metastases were more likely to respond [34]. These results and the observations in the present NSCLC study are similar in that patients with liver metastases are less likely experience tumor response, which is consistent with the immunosuppressive environment in the liver. The presence of lung lesions was not indicative of response in our cohort as opposed to the studies in melanoma, which is likely due to the presence of primary lung tumor in many of the advanced NSCLC patients. It is necessary to further investigate the similarities and differences of lesion-based responses across different tumor types, to understand if the differences are due to tumor microenvironment alone or are also related to the interactions between tumor and its microenvironment.

A limitation of the study includes the small number of patients treated at a single institution during the initial clinical experience studied with a retrospective design. However, such study design allowed us to report the evaluation of tumor response characteristics in NSCLC patients treated with commercially-prescribed nivolumab in a “real-world” setting, which has not been studied in detail despite an abundance of published data from prospective trials [4, 8, 9, 12]. Lack of molecular markers such as PD-L1 tissue staining in most patients in this clinical cohort limits the assessment of predictive values of such markers. Short follow-up time in this initial clinical experience also limits the evaluation of late responses and durable stability of the disease. These issues as well as the prognostic values of irRECIST1.1 assessments remain to be investigated with more mature follow-up data in a larger number of patients.

## Conclusion

In conclusion, the present study described tumor response characteristics in advanced NSCLC patients treated clinically with nivolumab monotherapy using irRECIST1.1, with a direct comparison with the conventional RECIST1.1 assessments. Response rate was identical between the two criteria, and no cases of pseudoprogression were observed during the treatment period of the study. Although a small percentage of pseudoprogressors were noted in the PD-1 inhibitor NSCLC trials, pseudoprogression appears to be a rare phenomenon in lung cancer. Inclusion of new lesions is a key component for future development of immune-related response evaluations to further understand their implications for treatment decisions both in trials and clinical practice. Responses of individual organs were different across anatomic organs, where adrenal lesions and lymph nodes were most responsive and liver lesions were least responsive to nivolumab. Further studies are needed to identify predictive and prognostic markers for immune-checkpoint inhibitor therapy and to address long-term effect of PD-1 inhibitor therapy on immune-related response characteristics in NSCLC.

## Methods

### Patients

The study population included 56 advanced NSCLC patients treated with nivolumab monotherapy at the Dana-Farber Cancer Institute after its FDA approval between March and August 2015 as a part of their standard clinical care. All patients had baseline CT prior to the initiation of nivolumab therapy, and at least one follow-up CT during therapy available for review. Nivolumab was administered at a dose of 3 mg/kg given intravenously every two weeks. Medical records and imaging studies were reviewed.

### Tumor burden measurements on the longitudinal scans

Baseline and all follow-up CT scans during nivolumab therapy were reviewed for the assessment of tumor burden by a board-certified radiologist (M.N.). Tumor burden was quantitatively assessed using irRECIST1.1 based on the previously published studies [19, 25, 26]. In brief, target lesions (≥10 mm in the longest diameter for non-nodal lesions and ≥15 mm in short axis for nodal lesions) were selected on baseline scans, allowing up to 2 lesions per organ and up to 5 lesions in total, as in RECIST1.1 [15–17]. Measurements of target lesions were performed on baseline and all follow-up CT scans during therapy. If new lesions appeared on the follow-up scans, the measurements of the new lesions were included in the sum of the measurements, as this has shown to be an important feature of immune-related response evaluations [18, 20, 25, 26]. Up to 2 new lesions per organ and 5 new lesions in total were allowed at each time point [20, 25]. New lesions had to be measurable (≥10 mm in the longest diameter for non-nodal lesions and ≥15 mm in short axis for nodes) to be included in the sum of the tumor measurements [25]. Measurable new lesions that were included in the measurements were recorded as “new target lesions”, and others lesions (i.e., non-measurable new lesions) were recorded as “non-target new lesions”. Other imaging studies such as brain MRI and PET/CT scans were also reviewed to identify new lesions and assess non-target lesions [35]. Conventional RECIST1.1 assessment was also performed. Follow-up scans were performed per treating providers’ discretion as a part of clinical care without predefined intervals.

### Tumor response assessment according to irRECIST1.1 and RECIST

Best overall response by irRECIST1.1 (irBOR) during therapy was assigned to each patient, using the thresholds of ≥30% decrease compared to baseline for partial response (PR) and ≥20% increase compared to nadir for progressive disease (PD), based on the prior studies showing the high concordance among the different methods of immune-related response evaluations [19, 25, 26]. Confirmation on 2 consecutive scans at least 4 weeks apart was required for irPD [18, 20, 25, 26]. Time to progression using irRECIST1.1 (irTTP) was obtained in each patient, allowing the inclusion of new lesion measurements and requiring confirmation of PD [25, 26]. As a comparison, BOR and TTP according to the conventional RECIST1.1 was also defined in each patient, where the appearance of new lesions or tumor burden increase ≥20% and 5 mm compared to baseline immediately defined PD without requiring confirmation [16, 17]. For both criteria, TTP was measured from the date of initiation of therapy to the date of progression as defined by the criteria. Unlike PFS, TTP does not include death as an event that defines the endpoint. Complete response (CR) required disappearance of all lesions, except for lymph nodes that need to be less than 10 mm in short axis, for both RECIST and irRECIST1.1 [18, 20, 25, 26].

### Lesion-based response assessment

Tumor measurements were further reviewed to assess responses of each target lesion. The same thresholds for response and progression for the total tumor burden (≥30% decrease for PR and ≥20% and 5 mm increase for PD) were applied for the lesion-based assessment. Each lesion was categorized according to the organs where it was located, including lung, lymph node, adrenal gland, liver, and other. Tumor size change in reference to baseline (%) and best response of each target lesion were compared across the anatomic organ categories.

### Statistical analysis

Comparison across groups was performed using a Fisher exact test for categorical variables and a Kruskal-Wallis test for continuous variables. TTP was estimated using a Kaplan-Meier method. All *p* values are based on a two-sided hypothesis. A *p* value of less than 0.05 was considered to be significant.

## Additional file

Additional file 1: The distribution of the number of target lesions according to the locations in patients grouped by irBOR. (TIF 7641 kb)

## Abbreviations

BOR: Best overall response
CR: Complete response
FDA: Food and Drug Administration
HPA: Hypothalamic–pituitary–adrenal
irRC: Immune-related response criteria
irRECIST1.1: Immune-related Response Evaluation Criteria In Solid Tumors1.1
NSCLC: Non-small-cell lung cancer
OS: Overall survival
PD: Progressive disease
PD-1: Programmed cell death-1
PD-L1: Programmed cell death ligand-1
PR: Partial Response
TTP: Time-to-progression
WHO: World Health Organization

## References

[1] Weber JS, D’Angelo SP, Minor D (2015). Nivolumab versus chemotherapy in patients with advanced melanoma who progressed after anti-CTLA-4 treatment (CheckMate 037): a randomised, controlled, open-label, phase 3 trial. Lancet Oncol.

[2] Robert C, Schachter J, Long GV (2015). Pembrolizumab versus Ipilimumab in Advanced Melanoma. N Engl J Med.

[3] Robert C, Long GV, Brady B (2015). Nivolumab in previously untreated melanoma without BRAF mutation. N Engl J Med.

[4] Rizvi NA, Mazieres J, Planchard D (2015). Activity and safety of nivolumab, an anti-PD-1 immune checkpoint inhibitor, for patients with advanced, refractory squamous non-small-cell lung cancer (CheckMate 063): a phase 2, single-arm trial. Lancet Oncol.

[5] Ribas A, Puzanov I, Dummer R (2015). Pembrolizumab versus investigator-choice chemotherapy for ipilimumab-refractory melanoma (KEYNOTE-002): a randomised, controlled, phase 2 trial. Lancet Oncol.

[6] Motzer RJ, Rini BI, McDermott DF (2015). Nivolumab for Metastatic Renal Cell Carcinoma: Results of a Randomized Phase II Trial. J Clin Oncol.

[7] Motzer RJ, Escudier B, McDermott DF (2015). Nivolumab versus Everolimus in Advanced Renal-Cell Carcinoma. N Engl J Med.

[8] Gettinger SN, Horn L, Gandhi L (2015). Overall Survival and Long-Term Safety of Nivolumab (Anti-Programmed Death 1 Antibody, BMS-936558, ONO-4538) in Patients With Previously Treated Advanced Non-Small-Cell Lung Cancer. J Clin Oncol.

[9] Garon EB, Rizvi NA, Hui R (2015). Pembrolizumab for the treatment of non-small-cell lung cancer. N Engl J Med.

[10] Topalian SL, Sznol M, McDermott DF (2014). Survival, durable tumor remission, and long-term safety in patients with advanced melanoma receiving nivolumab. J Clin Oncol.

[11] Topalian SL, Hodi FS, Brahmer JR (2012). Safety, activity, and immune correlates of anti-PD-1 antibody in cancer. N Engl J Med.

[12] Borghaei H, Paz-Ares L, Horn L (2015). Nivolumab versus Docetaxel in Advanced Nonsquamous Non-Small-Cell Lung Cancer. N Engl J Med.

[13] Herbst RS, Baas P, Kim DW, et al. Pembrolizumab versus docetaxel for previously treated, PD-L1-positive, advanced non-small-cell lung cancer (KEYNOTE-010): a randomised controlled trial. Lancet. 2015. 10.1016/S0140-6736(15)01281-7 26712084

[14] Brahmer J, Reckamp KL, Baas P (2015). Nivolumab versus Docetaxel in Advanced Squamous-Cell Non-Small-Cell Lung Cancer. N Engl J Med.

[15] Therasse P, Arbuck SG, Eisenhauer EA (2000). New guidelines to evaluate the response to treatment in solid tumors. European Organization for Research and Treatment of Cancer, National Cancer Institute of the United States, National Cancer Institute of Canada. J Natl Cancer Inst.

[16] Eisenhauer EA, Therasse P, Bogaerts J (2009). New response evaluation criteria in solid tumours: revised RECIST guideline (version 1.1). Eur J Cancer.

[17] Nishino M, Jagannathan JP, Ramaiya NH, Van den Abbeele AD (2010). Revised RECIST guideline version 1.1: What oncologists want to know and what radiologists need to know. AJR Am J Roentgenol.

[18] Nishino M, Jagannathan JP, Krajewski KM (2012). Personalized tumor response assessment in the era of molecular medicine: cancer-specific and therapy-specific response criteria to complement pitfalls of RECIST. AJR Am J Roentgenol.

[19] Nishino M, Tirumani SH, Ramaiya NH, Hodi FS (2015). Cancer immunotherapy and immune-related response assessment: The role of radiologists in the new arena of cancer treatment. Eur J Radiol.

[20] Wolchok JD, Hoos A, O’Day S (2009). Guidelines for the evaluation of immune therapy activity in solid tumors: immune-related response criteria. Clin Cancer Res.

[21] Lynch TJ, Bondarenko I, Luft A (2012). Ipilimumab in combination with paclitaxel and carboplatin as first-line treatment in stage IIIB/IV non-small-cell lung cancer: results from a randomized, double-blind, multicenter phase II study. J Clin Oncol.

[22] Hamid O, Robert C, Daud A (2013). Safety and tumor responses with lambrolizumab (anti-PD-1) in melanoma. N Engl J Med.

[23] Robert C, Ribas A, Wolchok JD (2014). Anti-programmed-death-receptor-1 treatment with pembrolizumab in ipilimumab-refractory advanced melanoma: a randomised dose-comparison cohort of a phase 1 trial. Lancet.

[24] Miller AB, Hoogstraten B, Staquet M, Winkler A (1981). Reporting results of cancer treatment. Cancer.

[25] Nishino M, Gargano M, Suda M, Ramaiya NH, Hodi FS (2014). Optimizing immune-related tumor response assessment: does reducing the number of lesions impact response assessment in melanoma patients treated with ipilimumab?. J Immunother Cancer.

[26] Nishino M, Giobbie-Hurder A, Gargano M, Suda M, Ramaiya NH, Hodi FS (2013). Developing a common language for tumor response to immunotherapy: immune-related response criteria using unidimensional measurements. Clin Cancer Res.

[27] Chiou VL, Burotto M (2015). Pseudoprogression and Immune-Related Response in Solid Tumors. J Clin Oncol.

[28] Caramella CAS, Facchinetti F, Massard C, Gazzah A, Planchard D, Soria JC, Besse B. Pseudo-progression in NSCLC with anti-PD-1/PD-L1 Antibodies: An Early Onset Event. RSNA. 2015;2015.

[29] Tagliamonte M, Petrizzo A, Tornesello ML, Ciliberto G, Buonaguro FM, Buonaguro L (2016). Combinatorial immunotherapy strategies for hepatocellular carcinoma. Curr Opin Immunol.

[30] Buonaguro L, Petrizzo A, Tagliamonte M, Tornesello ML, Buonaguro FM (2013). Challenges in cancer vaccine development for hepatocellular carcinoma. J Hepatol.

[31] Bornstein SR, Rutkowski H (2002). The adrenal hormone metabolism in the immune/inflammatory reaction. Endocr Res.

[32] Kanczkowski W, Sue M, Zacharowski K, Reincke M, Bornstein SR (2015). The role of adrenal gland microenvironment in the HPA axis function and dysfunction during sepsis. Mol Cell Endocrinol.

[33] Ribas A, Hamid O, Daud A (2016). Association of Pembrolizumab With Tumor Response and Survival Among Patients With Advanced Melanoma. JAMA.

[34] Goldinger SM, Tsai KK, Tumeh P, et al. Correlation between metastatic site and response to anti-Programmed Death-1 (PD-1) agents in melanoma. J Clin Oncol. 2016;34:(suppl; abstr 9549).

[35] Nishino M, Cardarella S, Jackman DM (2013). RECIST 1.1 in NSCLC patients with EGFR mutations treated with EGFR tyrosine kinase inhibitors: comparison with RECIST 1.0. AJR Am J Roentgenol.

