# Supplementary figures and images for: Immune-related response assessment during PD-1 inhibitor therapy in advanced non-small-cell lung cancer patients
Source: J Immunother Cancer. 2016 Dec 20;4:84. doi: 10.1186/s40425-016-0193-2 (PMC5168591; doi:10.1186/s40425-016-0193-2)

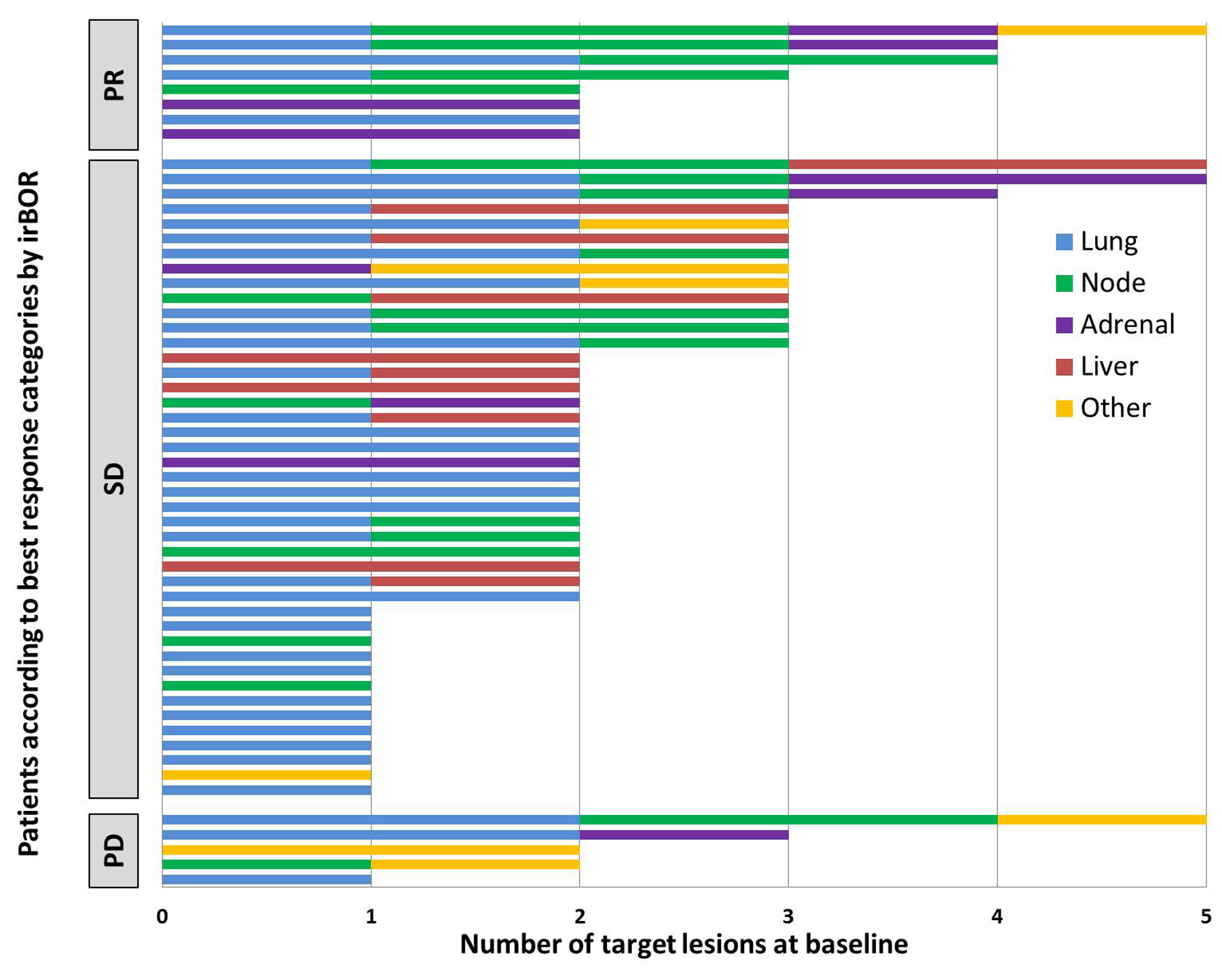

Supplement: Additional file 1: — The distribution of the number of target lesions according to the locations in patients grouped by irBOR. (TIF 7641 kb) [file 40425_2016_193_MOESM1_ESM.tif]
